# Quality of Reporting on Anastomotic Leaks in Colorectal Cancer Trials: A Systematic Review

**DOI:** 10.1097/DCR.0000000000003475

**Published:** 2024-08-07

**Authors:** Danique J.I. Heuvelings, Omar Mollema, Sander M.J. van Kuijk, Merel L. Kimman, Marylise Boutros, Nader Francis, Nicole D. Bouvy, Patricia Sylla

**Affiliations:** 1 NUTRIM School of Nutrition and Translational Research in Metabolism, Maastricht University, Maastricht, The Netherlands; 2 Department of Surgery, Maastricht University Medical Center, Maastricht, The Netherlands; 3 Department of Clinical Epidemiology and Medical Technology Assessment (KEMTA), Maastricht University Medical Centre, Maastricht, The Netherlands; 4 Department of Colon and Rectal Surgery, Cleveland Clinic Florida, Weston, Florida; 5 Division of Surgery and Interventional Science, University College, London, United Kingdom; 6 The Griffin Institute, Northwick Park and St. Mark’s Hospital, Harrow, United Kingdom; 7 GROW School for Oncology and Developmental Biology, Maastricht University, Maastricht, The Netherlands; 8 Division of Colon and Rectal Surgery, Icahn School of Medicine at Mount Sinai, New York, New York

**Keywords:** Anastomotic leakage, Colorectal surgery, Consensus, Reporting, Severity grading, Systematic review definitions

## Abstract

**BACKGROUND:**

Although attempts have been made in the past to establish consensus regarding the definitions and grading of the severity of colorectal anastomotic leakage, widespread adoption has remained limited.

**OBJECTIVE:**

A systematic review of the literature was conducted to examine the various elements used to report and define anastomotic leakage in colorectal cancer resections.

**DATA SOURCES:**

A systematic review was conducted using the PubMed, Embase, and Cochrane Library Database.

**STUDY SELECTION:**

All published randomized controlled trials, systematic reviews, and meta-analyses containing data related to adult patients undergoing colorectal cancer surgery and reporting anastomotic leakage as a primary or secondary outcome, with a definition of anastomotic leakage were included.

**MAIN OUTCOME MEASURES:**

Definitions of anastomotic leakage, clinical symptoms, radiological modalities and findings, findings at reoperation, and grading terminology or classifications for anastomotic leakage.

**RESULTS:**

Of the 471 articles reporting anastomotic leakage as a primary or secondary outcome, a definition was reported in 95 studies (45 randomized controlled trials, 13 systematic reviews, and 37 meta-analyses) involving a total of 346,140 patients. Of these 95 articles, 68% reported clinical signs and symptoms of anastomotic leakage, 26% biochemical criteria, 63% radiological modalities, 62% radiological findings, and 13% findings at reintervention. Only 45% (n = 43) of included studies reported grading of anastomotic leakage severity or leak classification, and 41% (n = 39) included a time frame for reporting.

**LIMITATIONS:**

There was a high level of heterogeneity between the included studies.

**CONCLUSIONS:**

This evidence synthesis confirmed incomplete and inconsistent reporting of anastomotic leakage across the published colorectal cancer literature. There is a great need to develop and implement a consensus framework for defining, grading, and reporting anastomotic leakage.

**REGISTRATION:**

Prospectively registered at PROSPERO (ID 454660).

Despite advances in preoperative risk assessment, operative techniques and strategies, and postoperative care, the incidence of anastomotic leakage (AL) after colorectal cancer (CRC) surgery has not improved over recent decades, with an incidence of 1.5% to 23% and with mortality rates as high as 16% to 29%.^1–5^ AL negatively impacts oncological outcomes, functional outcomes, and quality of life because of reoperation, permanent diversion, or delayed ostomy reversal.^2,3,5^ In addition, AL leads to increased hospital costs, which add to the overall economic burden associated with CRC surgery.^6^ AL can present as small defects without air or fluid extravasation or large defects with or without localized abscess, phlegmon, and/or peritonitis.^7,8^ The clinical impact of AL varies from minimal or no symptoms to substantial morbidity and mortality from abdominal and/or pelvic sepsis.^9^ Clinical studies where AL serves as a primary end point are often difficult to compare given considerable heterogeneity in the definition, severity grading, and diagnostic modalities used to assess AL.

Despite efforts to create a validated consensus definition and severity grading system by the International Study Group of Rectal Cancer (ISREC) in 2010, it has not been widely adopted in clinical practice.^10–12^ A survey study among Dutch and Chinese colorectal surgeons highlighted an ongoing lack of national and international agreement on definitions of AL.^13^ Hence, several definitions of AL continue to be used in studies, with the most controversy surrounding the radiological criteria considered diagnostic of AL. In 2020, a panel of 8 senior US surgeons attempted to reach a consensus on the definition of AL, specifically evaluating clinical and radiological criteria.^14^ Consensus could only be achieved in a few specific cases for both a radiological and clinical description and only for specific types of interventions.

The development of an internationally accepted standardized framework for defining, reporting, and grading colorectal AL is needed to facilitate earlier identification, reporting, and treatment of AL to reduce short- and long-term sequelae. A widely implemented standardized framework could serve as a template for clinical trials where the incidence of AL is used as a clinical end point. This systematic review (SR) aimed to gain insight into the different elements contributing to the general definition and reporting of AL in the literature. The findings of this study will serve as the basis of an ongoing project to develop a framework for reporting and grading AL after CRC surgery (Consensus Reporting of Colorectal Anastomotic Leaks).

## MATERIALS AND METHODS

This SR was reported according to the guidelines of the Preferred Reporting Items for Systematic Reviews and Meta-Analyses.^15^ The protocol has been prospectively registered at PROSPERO (ID 454660).

### Search and Information Sources

A literature search was performed on November 4, 2022, in the PubMed, Embase, and the Cochrane Library databases using MeSH, Emtree, and free terms (see Supplemental 1 at http://links.lww.com/DCR/C380). Reference lists of all publications were searched for additional relevant studies. The cross-referencing method was continued until no further relevant publications were identified.

### Selection Process

#### Inclusion and exclusion criteria

Randomized controlled trials (RCTs), SRs, and meta-analyses (MAs) containing data related to adult (older than 18 years) patients with CRC and in which AL was a primary or secondary outcome were considered eligible. Studies published before 2000 (the date of the first SR concerning AL definitions) were excluded, as were other publication types and articles not in English or Dutch. Articles were excluded if AL was not a primary or secondary outcome as stated in the methods section, no AL definitions were stated in the study, or patients were not undergoing oncological procedures.

#### Study selection

All search results were imported into a web tool designed for SRs (Rayyan).^16^ First, all duplicates were removed. Second, the screening of studies for eligibility was independently performed by 2 reviewers (D.J.I.H. and O.M.) using the predefined inclusion and exclusion criteria in 2 phases. In the first phase, articles were screened on the basis of title and abstract. Disagreements between reviewers were resolved by initial discussion to create consensus and/or by one of the senior authors (N.D.B.). As part of the second phase, full texts were assessed. If the eligibility criteria were found to be met after full-text screening by both reviewers, article inclusion followed. All references were stored in the EndNote reference management tool.

#### Data items and collection process

Two reviewers (D.J.I.H. and O.M.) independently extracted data from text, tables, and figures in a standardized, predefined datasheet. Data extraction for each article included first author, year of publication, country, study design, number of patients, number of studies in case of an SR or MA, study aims, surgical details, definitions or criteria used for AL assessment (clinical, biochemical, radiologic criteria or finding during reoperation), all definitions of AL, clinical symptoms associated with definitions of AL, radiological modalities and findings used in the diagnosis of AL, findings at reoperation for AL, as well as grading terminology or classifications for AL. We ensured definitions and reporting elements were not double-counted by cross-referencing RCTs included in SRs. When SRs provided their own AL definitions without detailing those from included studies, we treated these as separate entries. This method maintained data integrity. Data acquired through the outlined search strategy were summarized in tables.

#### Study risk-of-bias assessment

To assess the methodological quality of the included studies, the risk of bias was independently assessed by 2 reviewers (D.J.I.H. and O.M.). RCTs were assessed using the RoB2 tool, whereas (systematic) reviews and MAs were assessed using the ROBIS tool.^17,18^ All types of bias were evaluated and judged as low-, moderate-, or high-risk resulting in an overall bias judgment. The bias was visualized using the risk-of-bias visualization (Robvis) tool.^19^

## RESULTS

### Study Selection

**FIGURE 1.**
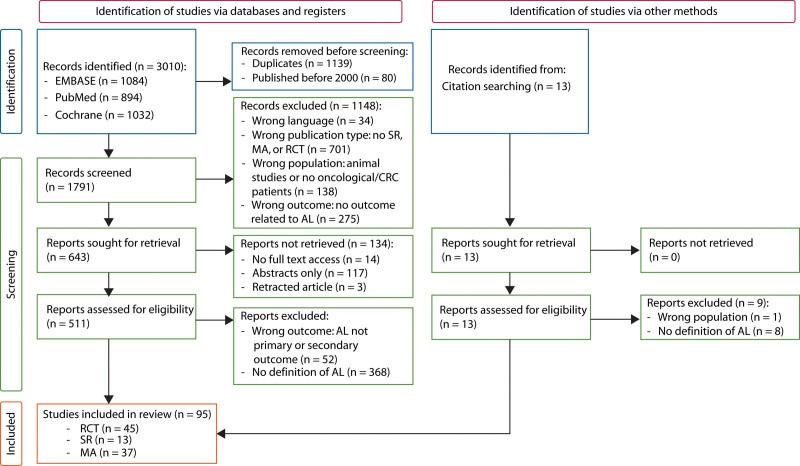
Flow diagram of inclusion process based on the PRISMA 2020 guidelines. AL = anastomotic leak; CRC = colorectal cancer; MA = meta-analysis; PRISMA = Preferred Reporting Items for Systematic Reviews and Meta-Analyses; RCT = randomized controlled trial; SR = systematic review.

The electronic search yielded 1792 studies after removing duplicates and publications before 2000. After screening abstracts, 644 potentially eligible studies remained on the basis of the predefined inclusion and exclusion criteria. Full-text assessment from 134 studies was not possible (ie, no full texts available, retracted articles), leaving 511 eligible articles. Reference checking resulted in 13 additional studies, resulting in 524 studies for full-text assessment. Fifty-three studies did not meet inclusion criteria; the remaining 471 studies reported AL as a primary or secondary outcome. Of these, 376 did not report a definition of AL, which resulted in the inclusion of 95 studies. The study selection process is summarized in Figure 1.

### Study Characteristics

The 95 studies included 45 RCTs, 13 SRs, and 37 MAs published between 2000 and 2022. The main characteristics of the included studies are summarized in Table 1.^12,20–113^

**TABLE 1.** Characteristics of included studies

*Author*	*Year*	*Country*	*Study design*	*No. of patients* ^a^	*No. of studies*	*Aim of the study*	*Type of resections included*
Alekseev et al^20^	2020	Russia	RCT	380	NA	To evaluate the usefulness of ICG in reducing AL in patients undergoing a stapled colorectal anastomosis	(L)AR with TME, left colectomy
Altomare et al^21^	2021	Italy	RCT	54	NA	To compare incidence of AL and severity of postoperative complications in patients undergoing LAR with diverting stoma or LAR with reinforcement of the anastomosis without diverting stoma	LAR with TME
Ansari et al^22^	2017	Australia	RCT	326	NA	To compare acute adverse events and postoperative complication rates in a randomized trial of short-course versus long-course preoperative radiotherapy	APR, (L)AR, Hartmann procedures
Badawi et al^23^	2015	Saudi Arabia	SR	6921	31	To review risk factors for and protective strategies against AL after minimally invasive surgery for CRC	(L)AR
Bakker et al^24^	2017	The Netherlands	RCT	402	NA	To evaluate the efficacy of the C-seal device in reducing AL after stapled colorectal anastomoses	All types of colorectal resections with stapled anastomoses
Balciscueta et al^25^	2020	Switzerland	SR and MA	1267	4	To evaluate the incidence of AL rate after laparoscopic rectal surgery after 1 vs 2 stapler firings for rectal transection	AR
Bao et al^26^	2022	Italy	RCT follow-up	311	NA	To evaluate overall survival, disease-free survival, and local and distant recurrence in patients with AL after LAR	LAR
Blanco-Colino et al^27^	2018	Spain	SR and MA	1302	5	To evaluate AL rates using ICG fluorescence imaging vs standard surgical care in CRC surgery	LAR with TME, right colectomy, left colectomy, sphincter-saving resection
Boelens et al^28^	2014	The Netherlands	RCT	123	NA	To investigate whether early enteral nutrition, as a bridge to a normal diet, can reduce postoperative ileus	LAR, APR, Hartmann procedure
Bretagnol et al^29^	2010	France	RCT	178	NA	To assess postoperative outcomes in patients undergoing sphincter-saving rectal resection for cancer without preoperative MBP	Mesorectal excision, sphincter-saving resection
Brisinda et al^30^	2009	Italy	RCT	77	NA	To compare surgical outcomes of end-to-end and end-to-side anastomosis after AR for T1–T2 rectal cancer	AR with TME or PME
Brown et al^31^	2001	Singapore	RCT	59	NA	To assess the effect of prophylactic drainage after LAR when anastomoses are located below the peritoneal reflection	LAR with total- or wide mesorectal excision
Bülow et al^32^	2006	Denmark	RCT	194	NA	To compare AL rates after AR with a loop ileostomy vs transanal stenting vs both vs neither	Anterior resection
Cong et al^33^	2015	China	SR	16,178	37	To evaluate AL requiring laparotomy and the associated rate of diverting stoma in initial AR for rectal cancer	(Ultra)LAR, sphincter-saving resection
Cong et al^34^	2014	China	SR and MA	24,232	39	To evaluate AL requiring reoperation and compare mortality in patients with AL relative to overall postoperative mortality after AR for rectal carcinoma	AR
Cong et a**l**^35^	2013	China	SR	24,288	70	To evaluate the pooled incidence and severity of AL and determine the average rate of AL for each grade after AR for rectal cancer	AR, (ultra)LAR, sphincter-saving resection
Maggiore et al^36^	2018	Egypt	RCT	57	NA	To compare the short-term operative as well as oncologic outcomes of robotic-assisted and laparoscopic rectal cancer resections	AR, (ultra)LAR, APR
Debakey et al^37^	2022	China	SR and MA	1556	7	To evaluate the TDT effect on AL prevention	Laparoscopic rectal resections
Deng et al^38^	2020	Italy	RCT	252	NA	To evaluate the usefulness of intraoperative assessment of anastomotic perfusion using ICG angiography in patients undergoing left-sided colon or rectal resection with colorectal anastomosis	LAR, left colectomy
Emile et al^39^	2022	Egypt	SR and MA	8786	27	To assess changes in surgical plan based on ICG fluorescence angiography on the rates of AL	All types of colorectal procedures
Finochi et al^40^	2020	France	MA	5115	12	To compare postoperative outcomes between patients undergoing rectal cancer resection performed by totally laparoscopic approach compared to those who underwent intraoperative conversion	APR, sphincter-saving resection
Floodeen et al^41^	2013	Sweden	RCT	45	NA	To compare patients with symptomatic AL after LAR for cancer diagnosed during the initial hospital stay with those in whom leakage was diagnosed after hospital discharge	LAR
Fujii et al^42^	2018	Japan	RCT	331	NA	To clarify whether the IMA should be tied at the origin (high tie) or distal to the left colic artery (low tie) in relation to AL	AR
Fujii et al^43^	2019	Japan	RCT subanalysis	331	NA	To determine whether the IMA should be tied at the origin (high tie) or distal to the left colic artery (low tie) in relation to AL	AR
Gadan et al^44^	2020	Sweden	RCT	232	NA	To investigate the incidence of and risk factors for permanent stoma beyond 5 y after LAR	LAR
Guenaga et a**l**^45^	2003	Brazil	SR	5805	18	To assess the safety and effectiveness of MBP based on morbidity and mortality after colorectal surgery	LAR
Ha et al^46^	2015	Korea	SR and MA	1118	6	To evaluate the effectiveness of transanal tube placement to prevent AL after LAR for rectal cancer using a stapling technique	LAR
Ha et al^47^	2017	South Korea	SR and MA	78,434	34	To assess the oncologic outcomes of AL after restorative surgery for CRC	All types of colorectal procedures
Habeeb et al^48^	2023	Egypt	RCT	74	NA	To compare outcomes of open colorectal anastomosis with side-to-end vs end-to-end configuration in nonemergent sigmoid and rectal cancer surgery in adults	(Ultra)LAR
Hajibandeh et al^49^	2019	UK	MA	436	4	To compare outcomes of temporary loop ileostomy closure during or after adjuvant chemotherapy after rectal cancer resection	LAR
He et al^50^	2022	China	RCT follow-up	203	NA	To analyze long-term impact of radiation on major LARS and permanent stoma rates	LAR
Hüser et al^51^	2008	Germany	SR and MA	2729	27	To evaluate the benefit of a defunctioning ileostomy or colostomy after LAR for CRC	LAR
Ivanov et al^52^	2011	Serbia	RCT	71	NA	To establish whether intraoperative air testing may reduce the dehiscence rate of stapled colorectal anastomoses	Sigmoid resection, LAR, sigmoidostomy diversion, Hartmann procedure
Jafari et al^53^	2021	USA	RCT	347	NA	To evaluate whether the use of fluorescence angiography to ensure anastomotic perfusion decreases AL after LAR	LAR
Karim et al^54^	2020	Switzerland	SR and MA	18,039	18	To evaluate cancer-specific outcomes after curative rectal cancer surgery comparing AL with no leak	All types of colorectal procedures
Kastora et al^55^	2021	UK	SR and MA	25,395	15	To assess whether NSAIDs, and their subcategories, increase AL in colonic anastomoses and to identify whether this affects specific anastomotic sites	Right hemicolectomy, left hemicolectomy, AR
Kelly et al^56^	2014	UK	SR and MA	14,344	19	To compare short-term and oncological outcomes following CRC resection performed by surgical trainees and expert surgeons	All types of colorectal procedures
Kim et al^57^	2022	Korea	SR and MA	1431	12	To compare the effects of high versus low IMA ligation in CRC surgery	(L)AR
Koedam et al^58^	2022	The Netherlands	RCT	1832	NA	To evaluate oncological outcomes with and without AL after CRC surgery	All types of colorectal procedures
Lee et al^59^	2018	Australia	SR and MA	1418	7	To evaluate the predictive value of cardiopulmonary exercise testing and field walk tests in surgical outcomes after CRC surgery	All types of colorectal procedures
Lin et al^60^	2021	China	SR and MA	3137	11	To investigate whether intraoperative ICG angiography can reduce the incidence of AL	LAR
Lindgren et al^61^	2011	Sweden	RCT follow-up	233	NA	To assess the risk for permanent stoma after LAR for rectal cancer	LAR
Lu et al^62^	2016	Australia	MA	13,655	11	To evaluate the best current evidence assessing AL in rectal cancer resections with curative intent and its impact on survival and cancer recurrence	All types of rectal procedures
Ma et al^63^	2020	China	SR and MA	3480	18	To assess the relationship between AL and long-term oncological outcomes after curative AR for rectal cancer	AR
Ma et al^64^	2019	China	RCT secondary analysis	125	NA	To quantify the changes in pelvic anatomic features caused by preoperative radiotherapy for CRC on pelvic MRI and evaluate the ability to predict AL	TME
Machado et al^65^	2003	Sweden	RCT	100	NA	To investigate functional outcomes of pouch vs nonpouch side-to-end anastomosis after standard TME surgery	LAR with TME
Mari et al^66^	2019	Italy	RCT	214	NA	To compare the incidence of genitourinary dysfunction and evaluate the incidence of AL and oncological outcomes in patients undergoing elective lap LAR + TME with either high or low ligation of the IMA	LAR with TME
Matsuda et al^12^	2015	Japan	RCT	100	NA	To clarify whether the level of ligation of the IMA in patients with rectal cancer affects defecatory function	AR
Matthiessen et al^67^	2007	Sweden	RCT	234	NA	To assess whether there is a difference in the rate of symptomatic AL in patients randomized to fecal deviation	LAR
McDermott et al^68^	2015	UK/Ireland	SR	–	451	To evaluate the role of preoperative, intraoperative, and postoperative factors in the development of colorectal AL	All types of colorectal procedures
Menahem et al^69^	2017	Germany	MA	660	3	To evaluate whether drainage of the extraperitoneal anastomosis after rectal surgery impacts the postoperative complication rate	Rectal resections
Mhatre et al^70^	2016		SR	20,441	–	To identify risk factors for AL and identify a standardized diagnostic protocol to reduce delay in diagnosis of AL	All types of colorectal procedures
Mrak et al^71^	2016	Austria	RCT	166	NA	To determine whether a protective diverting ileostomy reduces the AL rate	LAR
Neutzling et al^72^	2012	Brazil	SR	1233	9	To compare the safety and effectiveness of stapled and handsewn colorectal anastomosis. The following primary hypothesis was tested: the stapled technique is more effective because it decreases complications	All types of colorectal procedures
Oguz et al^73^	2007	Turkey	RCT	109	NA	To investigate the effect of l-alanine-l-glutamine on postoperative complication rate and duration of hospitalization in patients operated for CRC	All types of colorectal procedures
Okkabaz et al^74^	2017	Turkey	RCT	74	NA	To analyze the outcomes of J-pouch and side-to-end anastomosis in patients with rectal cancer treated with laparoscopic hand-assisted LAR	LAR
Pata et al^75^	2009	Belgium	SR with a MA and sensitivity analysis	4417	45	To determine whether a defunctioning stoma should be constructed routinely after TME or whether it could be used selectively to ensure patient safety	TME
Peeters et al^76^	2005	The Netherlands	Retrospective analysis of RCT	924	NA	To identify risk factors for symptomatic AL in patients undergoing TME for rectal cancer	TME
Peters et al^77^	2017	The Netherlands	RCT post hoc analysis	112	NA	To investigate the relationship between POI and inflammation and AL after CRC resection	All open colorectal resections
Podda et al^78^	2020	Italy	SR and MA	1120	4	To determine whether prophylactic drainage after colorectal anastomoses confers any advantage in the prevention and management of AL	All types of colorectal procedures
Pucciarelli et al^79^	2019	Italy	RCT	379	NA	To assess whether colonic J pouch reconstruction after LAR reduces the incidence of AL compared to standard straight colorectal anastomosis	LAR
Qi et al^80^	2022	China	SR and MA	580	8	To evaluate the predictive value of peritoneal fluid cytokines in the detection of AL after colorectal surgery	All types of colorectal procedures
Qu et al^81^	2015	China	SR and MA	4580	14	To quantify the clinicopathologic factors predictive for AL in patients undergoing laparoscopic AR for rectal cancer	Laparoscopic AR
Ren et al^82^	2021	China	RCT	64	NA	To provide a basis for evaluating the safety and effectiveness of laparoscopic TME	Laparoscopic TME
Rojas-Machado et al^83^	2016	Spain	SR and MA	–	68	To develop a new prognostic index to predict the risk of developing AL after CRC surgery	All types of colorectal procedures
Rolph et al^84^	2004	UK	Intervention review	903	3	To assess the effectiveness and safety of a prophylactic drain after elective colorectal anastomosis	All types of colorectal procedures
Rutkowski et al^85^	2014	Poland	RCT	177	NA	To evaluate the rate of local recurrence and distant recurrence in patients after R0 resection	TME
Saber et al^86^	2013	Egypt	RCT	156	NA	To evaluate the efficacy of tube cecostomy as an alternative to colostomy in the managing of patients with left-sided colonic carcinoma and rectal cancer with respect to postoperative morbidity and mortality and functional outcomes	All left colon or rectal cancer resections
Sangiorgio et al^87^	2021	Italy	Intervention review with MA	252	6	To systematically assess the efficacy of parenteral and oral antibiotic prophylaxis compared to parenteral-only prophylaxis for the prevention of SSI in patients undergoing laparoscopic surgery for CRC resection	All types of laparoscopic colorectal resections
Schardey et al^88^	2020	Germany	RCT	80	NA	To study the efficacy of topical antibiotic treatment on the incidence of AL in rectal cancer surgery	(L)AR
Selvamani et al^89^	2022	USA	SR	3451	12	To examine the need for blood markers that assist in the early diagnosis of AL after surgery	All types of colorectal procedures
Senagore et al^90^	2014	USA	RCT	258	NA	To assess whether the use of a synthetic, bioabsorbable staple line reinforcement material with circular staplers would reduce postoperative AL in patients with a colorectal, coloanal, or ileoanal anastomosis	All types of colorectal procedures
Shigeta et al^91^	2016	Japan	SR and MA	909	4	To evaluate the usefulness of a TDT for the prevention of AL after an AR for rectal cancer	AR
Singh et al^92^	2014	New Zealand	SR and MA	2483	7	To evaluate the predictive value of CRP in this setting	All types of colorectal procedures
Škrabec et al^93^	2022	Spain	SR	NA	9	To review and to assess the quality of the scientific articles regarding early and late AL after CRC surgery and their risk factors	All types of colorectal procedures
Snijders et al^94^	2012	The Netherlands	MA	10,343	22	To compare AL-related mortality in comparison to overall postoperative mortality after LAR for rectal cancer	LAR
Su’a et al^95^	2017	New Zealand	SR	8988	36	To assess biomarkers as potential diagnostic tests for preclinical detection of AL	All types of colorectal procedures
Su’a et al^96^	2020	New Zealand	MA	1639	8	To evaluate the accuracy of procalcitonin in the early diagnosis of AL after CRC surgery	All types of colorectal procedures
Tamura et al^97^	2021	Japan	RCT	161	NA	To assess the incidence of AL in patients with rectal cancer after laparoscopic AR with or without TDT on the hypothesis that it could contribute to prevent AL without reference to diverting stoma	LAR
Tan et al^98^	2009	Singapore	MA	11,429	25	To evaluate the need for routine stoma formation	LAR
Tocchi et al^99^	2000	Italy	RCT	112	NA	To investigate the role of omentoplasty, by means of intact omentum, in preventing AL after rectal resection	AR
Ulrich et al^100^	2009	Germany	RCT	34	NA	To evaluate the need for diverting ileostomy in patients undergoing LAR	LAR
Van’t Sand et al^101^	2011	The Netherlands	RCT subgroup analysis	63	NA	To evaluate the effects of MBP on morbidity and mortality after AL in elective colorectal surgery.	All types of colorectal procedures
Wang et al^102^	2017	China	SR and MA	11,535	14	To evaluate the impact of AL on disease recurrence and survival.	AR
Wang et al^103^	2016	China	SR and MA	909	4	To evaluate the efficacy of TDT placement after AR	AR
Whistance et al^104^	2013	UK	SR	NA	194	To summarize and undertake an in-depth analysis of outcome reporting in CRC surgery	All types of colorectal procedures
Wiggins et al^105^	2015	UK	SR and MA	2296	6	To compare the outcomes of GI anastomosis with and without the use of omentoplasty	All types of colorectal procedures
Wright et al^106^	2017	UK	SR	NA	13	To appraise the current evidence base into local biomarkers of AL allowing the identification of the most promising emerging biomarkers and discussion of their limitations and future potential clinical role	All types of colorectal procedures
Wu et al^107^	2014	China	MA	5612	11	To provide a comprehensive evaluation of the role of a protective stoma in LAR for rectal cancer	LAR
Xiao et al^108^	2011	China	RCT	398	NA	To investigate whether the use of a TDT as an alternative endoluminal diversion technique for rectal carcinoma can reduce the 30-d leakage rate after LAR	LAR
Yang et al^109^	2016	China	RCT	79	NA	To evaluate the anti-infectious effects of perioperative probiotics treatment in patients undergoing CRC resection	All types of colorectal procedures
Yang et al^110^	2019	China	SR and MA	8456	24	To evaluate the current scientific evidence of LCA nonpreservation versus LCA preservation in CRC surgery	All left colon or rectal cancer resections
Yeung et al^111^	2021	MA	MA	6647	23	To perform an MA of current CRP data in AL after colorectal surgery	All types of colorectal procedures
Zhang et al^112^	2016	China	MA	1803	11	To determine whether prophylactic placement of a drain in colorectal anastomosis can reduce postoperative complications	LAR
Zhao et al^113^	2021	China	RCT	560	NA	To assess the effect of TDT in AL prevention after laparoscopic LAR for rectal cancer	Laparoscopic LAR

AL = anastomotic leak; AR = anterior resection; APR = abdominoperineal resection; CRC = colorectal cancer; CRP = C-reactive protein; ICG = indocyanine green; IMA = inferior mesenteric artery; LAR = low anterior resection; LCA = left colic artery; MA = meta-analyses; MBP = mechanical bowel preparation; NA = not available; NSAID = non-steroidal anti-inflammatory drugs; PME = partial mesorectal excision; POI = postoperative ileus; RCT = randomized controlled trial; SR = systematic review; SSI = surgical site infection; TDT = transanal drainage tubes; TME = total mesorectal excision; USA = United States of America; UK = United Kingdom; – = authors did not report a total number of included patients or it was not clear to separate benign from malignant cases.

a Only malignant cases/patients after oncological resections.

### Risk of Bias in Studies

**FIGURE 2.**
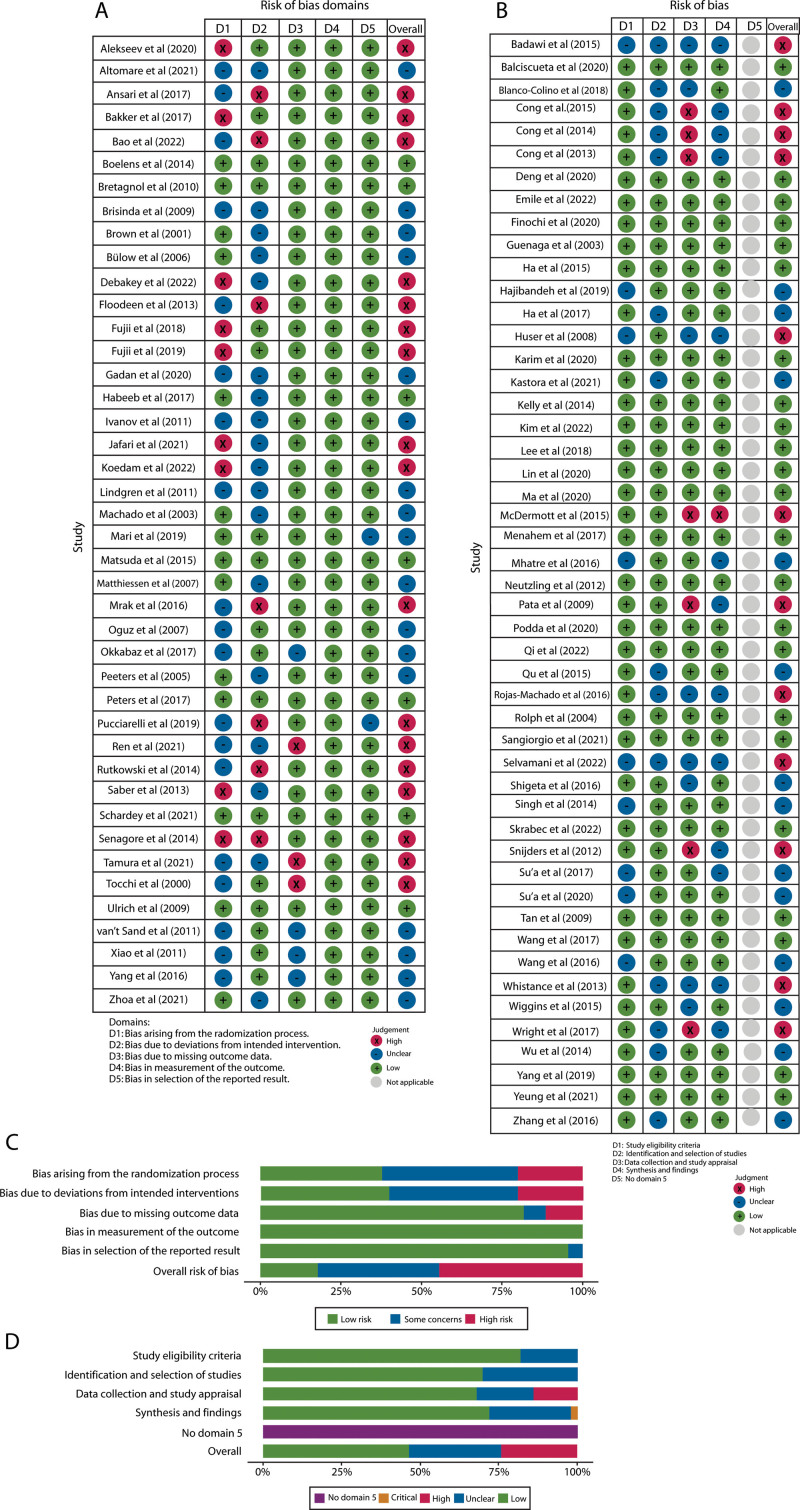
Risk-of-bias judgments. A, Risk of bias based on the RoB2 tool for RCTs and summary of the domain-level judgments for each study. B, Risk of bias based on the ROBIN tool for systematic reviews and meta-analyses and summary of the domain-level judgments for each study. C, Risk of bias judgments within each bias domain for RCTs. D, Risk of bias judgments within each bias domain for systematic reviews and meta-analyses. RCT = randomized controlled trial.

Forty-five RCTs (47%) were assessed for risk of bias (Figs. 2A and C). The judgment was based on the categories of bias arising from the randomization process, bias because of deviations from intended interventions, bias because of missing outcome data, bias in the measurement of the outcome, and bias in the selection of the reported results. On evaluation, the highest risk of bias was attributed to the randomization process and deviations from intended interventions. Nearly half of the studies (44%) were determined to have a high risk of bias.

Fifty SRs (53%) with or without MA were assessed for risk of bias (Figs. 2B and D). Risk assessment was based on study eligibility criteria, identification and selection of studies, data collection and study appraisal, and synthesis and findings. In general, these studies had a lower risk of bias than RCTs, with just a quarter of studies (24%) judged as having a high risk of bias.

### Terminology, Definitions, and Time Frame for AL Reporting

The term most frequently used to describe the complication of failure of the integrity of the anastomosis was AL. Other terms used less commonly included anastomotic dehiscence, insufficiency, failure, breakdown, defect, or separation. Nearly half of included studies (n = 44; Table 2; see Supplemental File 2 at http://links.lww.com/DCR/C380) used a more extensive definition to describe AL.^20,23,24,26,28,30,33,35,36,38,39,42,48,50,55,57,58,60,62–65,68,69,72,78,83,84,87–89,93–95,97–99,102–104,106,107,110,111^ The most commonly described definition was the ISREC definition (n = 25), which describes an AL as a defect of the intestinal wall at the anastomotic site (including suture and staple lines of neo-rectal reservoirs) leading to a communication between the intraluminal and extraluminal compartments. The time frame during which AL was diagnosed was reported in 39 studies, of which most (n = 24; 62%) reported AL-only within 30 days after index surgery.

**TABLE 2.** Overview of definitions and time frames used in the included studies

Definitions	N = 44/95 (46%)
A defect of the intestinal wall at the anastomotic site (including suture and staple lines of neorectal reservoirs) leading to a communication between the intraluminal and extraluminal compartments^a^	25 (57%)
Leak originating from staple/suture line	6 (14%)
Incontinuity at the anastomotic site detected clinically or radiologically within 30–60 d after surgery	3 (7%)
Anything other than a regular, uniform caliber at the level of the anastomosis	2 (5%)
Other definitions^b^	12 (27%)
Time frame of AL diagnosis (after surgery)	N = 39/95 (41%)
Within 7 d	1
Within 14 d	1
Within 30 d	24
>30 d	1
Within 90 d	2
Within 12 wk	1
Within 6 mo	1
Within hospital stay	2
No time limit reported (>6 mo)	4
Systematic review reports different times for all included articles	2

Percentages are calculated on the basis of number of publications reporting an element.

AL = anastomotic leak.

a Definition according to the International Study Group of Rectal Cancer.

b See Supplemental File 2 at http://links.lww.com/DCR/C380.

### Other Reporting Elements

An overview of all reporting elements is displayed in Table 3.

**TABLE 3.** Overview of reported elements subdivided in clinical, biochemical, imaging, reinterventions, and grading terms

*Reporting element*	*No. of publications*
Clinical signs and/or symptoms	N = 65/95 (68%)
Discharge from the drain	51 (78%)
Peritonitis	42 (65%)
Fever	25 (38%)
Fistula formation (eg, rectovaginal fistula)	23 (35%)
Discharge from the wound	17 (26%)
Local physical examination (eg, bowel obstruction, gastric retention, facial dehiscence, abdominal pain)	14 (22%)
Anastomotic dehiscence/defect	11 (17%)
Discharge of pus per rectum	8 (12%)
Sepsis	8 (12%)
Cardiac complications (eg, atrial fibrillation, tachycardia)	5 (8%)
Deterioration of clinical condition	3 (5%)
Tachypnea	3 (5%)
Decreased urine production	3 (5%)
Mental status change (eg, agitation, lethargy)	3 (5%)
Nutritional status (eg, tube feeding, total parental nutrition)	3 (5%)
Diarrhea	1 (2%)
Organ failure	1 (2%)
Abdominal distention	1 (2%)
Biochemical elements	N = 25/95 (26%)
Leukocytosis/white cell count	22 (88%)
CRP elevation	7 (28%)
Worsening of renal function (eg, creatinine, urea)	3 (12%)
Increase of procalcitonin	2 (8%)
Leukopenia	1 (4%)
pH changes	1 (4%)
Lactate (increase)	1 (4%)
Pyruvate (increase)	1 (4%)
Cytokines (increase)	1 (4%)
Lysozymes (increase)	1 (4%)
Matrix metalloproteinases (increase)	1 (4%)
Culture of intra-abdominal bacteria	1 (4%)
Other postoperative inflammatory markers (ie, I-FABP, TNFRSF1A, IL-6, IL-8, CCL2)	1 (4%)
Modality	N = 60/95 (63%)
CT scan	
Not specified	36 (60%)
With contrast (not specified)	6 (10%)
With contrast enema	6 (10%)
With IV contrast	1 (2%)
With oral contrast	1 (2%)
Endoscopy	
Not specified	13 (22%)
Sigmoidoscopy	11 (18%)
Rectoscopy	5 (8%)
Proctoscopy	2 (3%)
Colonoscopy	1 (2%)
Enteroscopy	1 (2%)
Unspecified contrast studies	
Contrast enema	20 (33%)
Water soluble contrast enema	7 (12%)
Radiological contrast study	3 (5%)
Water soluble contrast study	2 (3%)
X-ray	
With contrast (eg, not specified, water soluble)	5 (8%)
With contrast enema (eg, not specified, water soluble)	4 (7%)
Not specified	1 (2%)
Fluoroscopy	
Gastrografin enema	4 (7%)
Ultrasound	3 (5%)
MRI	2 (3%)
PET	1 (2%)
Imaging findings	N = 59/95 (62%)
Abdominal or pelvic collection/abscess in the proximity of the anastomosis	54 (92%)
Extravasation of contrast	16 (27%)
Presence of fluid/air around the anastomosis	9 (15%)
Anastomotic dehiscence/breakdown of any staple line/anastomotic defect	10 (17%)
Fistula formation (eg, rectovaginal fistula)	9 (15%)
Fecal peritonitis	1 (2%)
Abscess with a communication to the anastomosis	1 (2%)
Reintervention findings	N = 12/95 (13%)
Evidence of an anastomotic defect or dehiscence	9 (75%)
Fistula formation	3 (25%)
Postoperative peritonitis	2 (17%)
Air, fluid, GI contents, or contrast material	1 (8%)
Pericolic abscess or phlegmon	1 (8%)
Pelvic, intra-abdominal, or retroperitoneal abscess	1 (8%)
Generalized purulent peritonitis	1 (8%)
Generalized fecal peritonitis	1 (8%)
Grading terms	N = 43/95 (45%)
ISREC classification	21 (49%)
Other classifications	
Clavien-Dindo	8 (19%)
Hinchey	1 (2%)
Major vs minor leaks	6 (14%)
Radiological vs clinical leaks	4 (9%)
Clinical vs subclinical leaks	4 (9%)
Generalized vs localized leaks	1 (2%)
Early vs late leaks	1 (2%)
Significant vs nonsignificant leaks	1 (2%)
Complete vs partial leaks	1 (2%)

CCL2 = C-C motif chemokine ligand 2; CRP = C-reactive protein; I-FABP = intestinal fatty acid–binding protein; IL-6 = interleukin-6; IL-8 = interleukin-8; ISREC = International Study Group of Rectal Cancer; TNFRSF1A = tumor necrosis factor receptor superfamily member 1A.

### Clinical and Biochemical Elements

A total of 65 studies (68%) reported clinical signs and symptoms associated with AL, either as part of the formulated definition or in the description of the method of diagnosis.^20–22,25–27,29,31,32,34,36–46,49–53,58,61–64,67–78,80–82,84–86,90–95,97–99,101,103,104,106,108,110,112,113^ The most frequently described clinical signs/symptoms were purulent or feculent discharge from a drain, peritonitis, fever, and fistula formation. In addition, 26% of publications (n = 25) reported biochemical elements in the description of the method of AL diagnosis.^25,31,34,36,40,51,52,64,68–71,77,78,81,93,95,99,101,104,108,111–113^ The most described biochemical markers were leukocytosis and C-reactive protein.

### Radiological Modalities and Elements

Radiological modalities were specified in 63% of publications (n = 60).^20–22,25–31,36,38–44,46,50,53,58,61,63,64,66–71,73–81,84–86,88,90–92,94–96,98,99,101,104–106,108,110,112,113^ Most authors confirmed the suspicion of AL by a CT scan. In more than half of the studies, the authors did not specify whether the CT scan was performed with or without oral or rectal contrast. If specified, most of them used contrast enemas. Besides CT scans, endoscopic studies (eg, sigmoidoscopy, rectoscopy) were used to assess AL. Other modalities used included x-ray with or without contrast, gastrografin enema, ultrasound, MRI, and PET. An abdominal or pelvic collection and/or abscess in the proximity of the anastomosis was the most frequently described imaging finding when diagnosing a leak. Extravasation of contrast, the presence of air or fluid around the anastomosis, descriptions of anastomotic dehiscence, breakdown of any staple line, and anastomotic defect were also used.

### Reoperations

Findings at reoperation were described in 13% of the included publications (n = 12).^22,27,28,53,66,75,77,92,94,96,101,104^ The most frequently reported finding was the visualization of anastomotic dehiscence and/or anastomotic defect at the time of reoperation. Other findings at reoperation were fistula formation and postoperative peritonitis.

### AL Severity Grading Systems

Grading or classification of AL severity was reported in 45% of included studies (n = 43).^12,20,21,23,26,28,29,33–36,38,39,41–43,47,49,50,56,57,60,64,66,68,69,72,73,77,79,83,84,88,90,92–95,97,99,101,112,113^ Nearly half of publications used the ISREC grading system. This classification ranks AL into 3 grades (grade A, B, or C) based on clinical management.^10^ Clavien-Dindo grading was used in 19% of publications (n = 8).^28,29,36,42,43,57,77,94^ Leaks were classified as major versus minor leaks in 14% of the articles (n = 6), whereas radiological versus clinical and clinical versus subclinical leaks were reported in 4 articles.^21,49,64,66,69,72,73,79,92,94,96,99,104,112^

## DISCUSSION

This SR aimed to evaluate the various elements and criteria used to report on the definition and grading of colorectal AL after CRC resections. This current literature review reveals the lack of a widely accepted and applied definition of colorectal AL. Despite the increase in the number of a high level of evidence publications (RCTs, SRs, and MAs) on this topic in recent years, 72% of publications (n = 376) screened for eligibility did not include a specific definition to assess the presence of AL, although the incidence of AL served as a primary or secondary outcome. Based on our literature search, only 18% of eligible studies (n = 95) specified how AL was defined. To gain knowledge of general definitions of AL across eligible publications, specific elements contributing to the definition and grading of the severity of leaks were compared across studies when applicable (ie, clinical, biochemical- and radiological findings, findings at reoperation, severity grades). The latter led to another noteworthy finding: the extensive range of elements used led to vast variations in the reported colorectal AL rates (based on the various categories or domains used) and ultimately resulted in difficulty comparing findings across studies.

Overall, to support the diagnosis of an AL, clinical signs and symptoms were used in 68% of included studies, radiological modalities and radiological findings in 63% and 62%, respectively, biochemical elements in 26%, and findings at reoperation only in 13% of studies. In addition, 45% of studies reported grading the severity of AL, with 46% reporting a more detailed definition and 41% including a time frame for AL reporting.

A consensus study by van Helsdingen et al^13^ previously reported recommendations for a definition and category elements of AL based on experts’ opinions. By comparing the results of our review to the recommendations formulated in this consensus, we confirm a lack of reporting the categories suggested (clinical parameters, laboratory tests, radiological findings, findings during reoperation, grading systems, timing, and location of the tumor). The most common element used for AL reporting was clinical symptoms and signs associated with AL. Compared to the ISREC definitions, our results for clinical elements showed many similarities. However, several clinical elements from our search were not included in the original ISREC classification.^10^ The most frequently used biochemical result was leukocytosis. In contrast, although C-reactive protein was also included in the ISREC classification, its use was only mentioned in 7 studies.^36,64,68,70,77,95,111^ There is no uniformity in recommendations regarding a preferred imaging modality when suspecting an AL. The most often used modality to support the diagnosis of a leak in our analysis was CT. However, whether these were CTs performed with rectal, intravenous, or oral contrast was often unclear. Although a previous SR and MA by Kornmann et al^114^ reported the scarce and poor quality of evidence regarding the predictive value of CT in diagnosing AL, Matsuda et al^12^ and Lim et al^8^ specifically used CT for confirmation when there was suspicion of AL. It is unclear how much additional information rectal contrast provides over clinical assessment for low anastomoses.^115^ Notably, the role of endoscopic assessment in assessing AL is poorly investigated, despite low procedural risk and rapid detection of AL.^116^ Besides the type of imaging modality used, the detailed findings are important, too. The most frequently described finding was an abdominal or pelvic collection and/or abscess in the proximity of the anastomosis on CT scan, although radiological criteria considered diagnostic of AL remain controversial.^14^ On diagnosis of AL, the type of reintervention and findings at reintervention were underreported in the summarized evidence. It is important to report the type of reintervention(s) as this may correlate with time to resolution of AL, return to function, and long-term outcomes and quality of life. Only 13% of included studies reported type of reintervention(s), which highlights a significant gap in reporting.

The lack of standardized definitions and agreement on the specific elements of an AL contributed to significant variations in the reported rates, making it challenging to identify risk factors for leaks and evaluate the effectiveness of specific therapeutic and prophylactic interventions. Most studies considered AL to involve a breach in the integrity of the intestinal wall at the site of colorectal or coloanal anastomosis, with severity ranging from incidental findings to life-threatening sepsis requiring further surgery. However, substantial variability was uncovered regarding the minimum criteria for reporting AL.

Grading the severity of AL may have major implications with respect to timing and type of required intervention, prognosis, and short- and long-term outcomes. However, fewer than half of the included studies reported grading or classification of AL. The most common grading system reported was the ISREC classification, followed by the Clavien-Dindo classification, although this is not specific to AL.^117^ Furthermore, our results also showed that there was some effort toward classifying leaks based on the degree of clinical severity (ie, significant vs nonsignificant leaks, clinical vs radiological leaks); however, the specific terminology used was ill defined and nonstandardized. One important attribute that may play an important role in reporting and managing ALs is the time frame in which AL is identified, with a clear distinction between early versus late or delayed leaks. Our review found that the time frame of leak diagnosis, that is, early and late or delayed, was only reported in 1 article,^41^ and most other studies described a 30-day postoperative time frame for reporting. Including early and late time frames as an element in the standardized reporting of AL may prevent underreporting of late/delayed leaks and their sequelae, facilitate earlier management, and improve long-term outcomes.

The stigma associated with leaks and the use of institutional AL rates as a measure of surgical quality may contribute to the generalized reluctance to investigate leaks early and consistently, as reflected in the wide range of reported diagnostic elements in our review. This stigma must be balanced against the potential benefits of adopting a standardized reporting framework that facilitates earlier diagnosis, management, and resolution of leaks. Also, within current reporting systems like the National Surgical Quality Improvement Program, the reporting of an AL is presently contingent on the specific intervention undertaken and lacks background information (this encompasses a spectrum of scenarios: instances where no documented treatment intervention is recorded, cases managed through interventional methods, situations addressed with noninterventional or nonoperative approaches, instances necessitating reoperation, situations where there is no definitive diagnosis of a leak or a leak-related abscess, and cases categorized as unknown). The need for standardized, well-accepted terminology for reporting AL remains an important issue, especially when evaluating the effectiveness of targeted interventions and/or comparing procedural outcomes. Several issues need to be addressed before formulating a novel framework for reporting and grading colorectal AL that will gain wide acceptance. A consensus agreement needs to be reached with respect to which clinical, radiologic or endoscopic, and/or biochemical elements are most suggestive of AL, as reporting rates of these elements vary widely. Second, agreement is also needed with respect to grading the severity of leaks, which may take into account not only the type of intervention(s) required but also short- and long-term sequelae and impact on patients. Third, additional elements relevant to the time frame of diagnosis and management of leaks should be routinely incorporated in reporting, with a clear distinction between early versus late/delayed AL diagnosis. Finally, additional features of AL, with potential implications on outcomes and interventions, may need to be included, such as anastomotic height and protective fecal diversion.

There are some limitations of the current work. The heterogeneity between the included studies and varying data presentations prohibited a more detailed analysis. Also, not all papers solely reported on oncological cases. Furthermore, a deliberate choice was made only to include high-level evidence publications (ie, RCTs and SRs with or without MAs). However, based on the findings of these studies, the urgency of achieving uniformity in the reporting and grading of colorectal AL is highlighted. This uniform process would facilitate quality assurance in reporting diagnostic elements, enable transparency of study results, and provide a reliable interpretation of MAs. The development of a general outcome AL set may be helpful in tackling further reporting gaps. Consequently, the findings of this study may inform the development of a consensus framework for the reporting and grading of AL after CRC surgery.

## CONCLUSIONS

This SR highlights substantial heterogeneity in the elements used to define colorectal AL across high-level evidence literature, reflecting the need for a widely accepted framework that can guide the definition, grading, and reporting of AL. Standardized reporting of AL is essential for mitigating delays in diagnosis and treatment, promoting the development of treatment guidelines, and addressing existing shortcomings.

## ACKNOWLEDGMENTS

The authors would like to thank Gregor Franssen, who was involved as a professional clinical librarian to ensure an appropriate search strategy.

**CoReAL Collaborative:** Michel Adamina, Alberto Arezzo, Mahdi Al-Taher, Tan Arulampalam, Saba Balvardi, Himani Bhatt, Marta Botti, Stephanie O. Breukink, David A. Clark, Freek Daams, Jennifer S. Davids, Anse De Sadeleer, Abe Fingerhut, Zoe Garoufalia, Anke H.C. Gielen, Mukesh G. Harisinghani, Roel Hompes, Neil H. Hyman, Mehraneh D. Jafari, John T. Jenkins, Audrey C.H.M. Jongen, Deborah S. Keller, Samuel H. Lai, Jérémie H. Lefevre, Bibi Martens, Justin A. Maykel, Jeongyoon Moon, Nariaki Okomoto, Ian Paquette, Gianluca Pellino, Sherief F. Shawki, Benjamin D. Shogan, Chelliah Selvasekar, Simon Siu-Man Ng, Jasper Stijns, Patricia Tejedor, William Tzu-Liang Chen, Yu-Ting T van Loon, Christiaan van Der Leij, Steven D. Wexner, Elizabeth Wick, and Marina Yiasemidou.

## Supplementary Material


