# Supplementary material for: Quality of Reporting on Anastomotic Leaks in Colorectal Cancer Trials: A Systematic Review
Source: Dis Colon Rectum. 2024 Aug 7;67(11):1383–401. doi: 10.1097/DCR.0000000000003475 (PMC11477855; doi:10.1097/DCR.0000000000003475)
Supplement: Supplementary file 1 [file dcr-67-1383-s001.pdf]

SUPPLEMENTARY

Supplementary 1 – Search strategy

Database	Search Syntax
**Pubmed**	(((("Colorectal Neoplasms"[MeSH] OR (("Neoplasms"[MeSH] OR carcinoma*[tiab] OR adenocarcinoma*[tiab] OR neoplas*[tiab] OR tumour*[tiab] OR tumor*[tiab] OR oncolog*[tiab] OR malignan*[tiab] OR cancer*[tiab]) AND (colorectal*[tiab] OR colon[tiab] OR colonic[tiab] OR rectal[tiab] OR rectum[tiab] OR sigmoid*[tiab]))) AND ("Colectomy"[MeSH] OR "Colorectal Surgery"[MeSH] OR "Rectum/surgery"[MeSH] OR "Colon/surgery"[MeSH] OR ((large bowel[tiab] OR colorectal*[tiab] OR colon[tiab] OR rectum[tiab] OR rectal[tiab] OR ileocaecal[tiab] OR caecum[tiab] OR low anterior[tiab]) AND (resection*[tiab] OR surg*[tiab] OR anastomo*[tiab] OR "Anastomosis, Surgical"[MeSH])) OR (colectom*[tiab] OR hemicolectom*[tiab] OR "total mesorectal excision*"[tiab] OR proctocolectom*[tiab] OR "abdominal perineal resection*"[tiab]))) AND ("Anastomotic Leak"[MeSH] OR (anastomo*[tiab] AND ("adverse effects"[Subheading] OR "complications"[Subheading] OR leak*[tiab] OR complication*[tiab] OR defect*[tiab] OR separation*[tiab] OR dehiscence*[tiab] OR breakdown*[tiab] OR abscess*[tiab]))) AND (((systematic review[pt] OR (((systematic review[ti] OR systematic literature review[ti] OR systematic scoping review[ti] OR systematic narrative review[ti] OR systematic qualitative review[ti] OR systematic evidence review[ti] OR systematic quantitative review[ti] OR systematic meta-review[ti] OR systematic critical review[ti] OR systematic mixed studies review[ti] OR systematic mapping review[ti] OR systematic cochrane review[ti] OR systematic search and review[ti] OR systematic integrative review[ti]) NOT comment[pt] NOT (protocol[ti] OR protocols[ti])) NOT MEDLINE [subset]) OR (Cochrane Database Syst Rev[ta] AND review[pt])) OR ("Meta-Analysis"[pt] OR meta analysis[ti])) OR ("Randomized Controlled Trial"[pt] OR "Controlled Clinical Trial"[pt] OR ((random*[tiab] AND (controlled[tiab] OR control[tiab] OR placebo[tiab] OR versus[tiab] OR vs[tiab] OR group[tiab] OR groups[tiab] OR comparison[tiab] OR compared[tiab] OR crossover[tiab] OR cross-over[tiab]) AND (trial[tiab] OR study[tiab])) OR ((single[tiab] OR double[tiab] OR triple[tiab]) AND (masked[tiab] OR blind*[tiab])))))) NOT ((("Animals"[MeSH]) OR "Models, Animal"[MeSH] NOT "Humans"[MeSH]) NOT (letter[pt] OR comment[pt] OR editorial[pt]))

**Embase**	(exp colorectal tumor/ or exp colorectal cancer/ or ((neoplasm/ or (carcinoma* or adenocarcinoma* or neoplas* or tumour* or tumor* or oncolog* or malignan* or cancer*).ti,ab,kw.) adj3 (colorectal* or colon or colonic or rectal or rectum or sigmoid*).ti,ab,kw.)) and (exp colorectal surgery/ or exp rectum surgery/ or exp colon surgery/ or ileoanal anastomosis/ or ileorectal anastomosis/ or ((large bowel or colorectal* or colon or rectum or rectal or ileocaecal or caecum or low anterior).ti,ab,kw. adj3 (resection* or surg* or anastomo*).ti,ab,kw. or (colectom* or hemicolectom* or "total mesorectal excision*" or proctocolectom* or "abdominal perineal resection*").ti,ab,kw.)) and (postoperative complication/su or exp anastomosis leakage/ or anastomosis/co or (anastomo* adj3 (leak* or complication*)).ti,ab,kw.) and ((("systematic review"/ or (systematic review.ti. or systematic literature review.ti. or systematic scoping review.ti. or systematic narrative review.ti. or systematic qualitative review.ti. or systematic evidence review.ti. or systematic quantitative review.ti. or systematic meta-review.ti. or systematic critical review.ti. or systematic mixed studies review.ti. or systematic mapping review.ti. or systematic cochrane review.ti. or "systematic search and review".ti. or systematic integrative review.ti.)) or (meta analysis/ or meta analysis.ti.) or (randomized controlled trial/ or ((random*.ti,ab. and (controlled.ti,ab. or control.ti,ab. or placebo.ti,ab. or versus.ti,ab. or vs.ti,ab. or group.ti,ab. or groups.ti,ab. or comparison.ti,ab. or compared.ti,ab. or crossover.ti,ab. or cross-over.ti,ab.) and (trial.ti,ab. or study.ti,ab.)) or ((single.ti,ab. or double.ti,ab. or triple.ti,ab.) and (masked.ti,ab. or blind*.ti,ab.))))) NOT ((exp animal/ or nonhuman/) NOT exp human/) NOT (letter or editorial).pt.)
**Cochrane**	("Colorectal Neoplasms"[MeSH] OR (("Neoplasms"[MeSH] OR carcinoma*:ti,ab,kw OR adenocarcinoma*:ti,ab,kw OR neoplas*:ti,ab,kw OR tumour*:ti,ab,kw OR tumor*:ti,ab,kw OR oncolog*:ti,ab,kw OR malignan*:ti,ab,kw OR cancer*:ti,ab,kw) AND (colorectal*:ti,ab,kw OR colon:ti,ab,kw OR colonic:ti,ab,kw OR rectal:ti,ab,kw OR rectum:ti,ab,kw OR sigmoid*:ti,ab,kw))) AND ("Colectomy"[MeSH] OR "Colorectal Surgery"[MeSH] OR "Rectum/surgery"[MeSH] OR "Colon/surgery"[MeSH] OR ((large bowel:ti,ab,kw OR colorectal*:ti,ab,kw OR colon:ti,ab,kw OR rectum:ti,ab,kw OR rectal:ti,ab,kw OR ileocaecal:ti,ab,kw OR caecum:ti,ab,kw OR low anterior:ti,ab,kw) AND (resection*:ti,ab,kw OR surg*:ti,ab,kw OR anastomo*:ti,ab,kw OR "Anastomosis, Surgical"[MeSH])) OR (colectom*:ti,ab,kw OR hemicolectom*:ti,ab,kw OR "total mesorectal excision*":ti,ab,kw OR proctocolectom*:ti,ab,kw OR "abdominal perineal

	resection*":ti,ab,kw)) AND ("Anastomotic Leak"[MeSH] OR (anastomo*:ti,ab,kw AND ("adverse effects"[Subheading] OR "complications"[Subheading] OR leak*:ti,ab,kw OR complication*:ti,ab,kw OR defect*:ti,ab,kw OR separation*:ti,ab,kw OR dehiscence*:ti,ab,kw OR breakdown*:ti,ab,kw OR abscess*:ti,ab,kw))

Supplementary 2 – All theoretical definitions formulated by included studies

**Theoretical definition**	**Reported in papers**: N = 44
A defect of the intestinal wall at the anastomotic site (including suture and staple lines of neo-rectal reservoirs) leading to a communication between the intra- and extra-luminal compartments.*	25
As staple line leaks both of the circular and linear stapler.	1
An incontinuity at the anastomosis detected clinically or radiologically.	1
The definition of AL has been broadly agreed to encompass a breach of the surgical join between two hollow viscera which may lead to an observable leak of luminal contents.	1
Anastomotic leakage was defined as incontinuity at the anastomotic site detected clinically or radiologically within 30/60 days after surgery.	3
AL is defined as the connection between the cavity and the outside of the cavity caused by defects in the integrity of the intestinal wall at the anastomosis.	1
A breach in a surgical join between two hollow viscera, with or without active leak of luminal contents.	1
Pelvic sepsis adjacent to an anastomosis, even if no communication with the bowel lumen could be demonstrated, was considered to have originated from a leak.	1
A pelvic sepsis: Clinical staple line leak, infectious collections in the pelvis, with or without proven staple line leak and entrovaginal fistulas.	1
Defective anastomosis with signs of abscess formation.	1
Anything other than a regular, uniform caliber at the level of the anastomosis.	2
Radiological or operative evidence of defect in the enteric wall at the site of the anastomosis.	1
Unequivocal clinical evidence of an intestinal wall breakdown at the anastomotic site, with or without radiological, endoscopic, or surgical confirmation.	1
Leak originating from staple/suture line.	6
Radiological anastomotic dehiscence: presence of a leak with the control postoperative enema in a patient who had no evidence of clinical anastomotic leak.	1
generalised (gross abdominal faecal contamination) or local (localised faecal contamination in the peri-anastomotic space).	1

** According to the International Study Group of Rectal Cancer.*
